# Supplementary material for: Higher order genetic interactions switch cancer genes from two-hit to one-hit drivers
Source: Nat Commun. 2021 Dec 3;12:7051. doi: 10.1038/s41467-021-27242-3 (PMC8642467; doi:10.1038/s41467-021-27242-3)
Supplement: Supplementary file 1 — Supplementary information [file 41467_2021_27242_MOESM1_ESM.pdf]

Supplementary Information for

Higher order genetic interactions switch cancer genes from two-hit to one-hit drivers

Park et al.

Supplementary Tables
Supplementary Figures

Supplementary Tables

Supplementary Table 1. List of cancer types

Study Abbreviation	Study Name
ACC	Adrenocortical carcinoma
BLCA	Bladder Urothelial Carcinoma
BRCA	Breast invasive carcinoma
CESC	Cervical squamous cell carcinoma and endocervical adenocarcinoma
CHOL	Cholangiocarcinoma
COADREAD	Colon Rectum adenocarcinoma
DLBC	Lymphoid Neoplasm Diffuse Large B-cell Lymphoma
ESCA	Esophageal carcinoma
GBM	Glioblastoma multiforme
HNSC	Head and Neck squamous cell carcinoma
KICH	Kidney Chromophobe
KIRC	Kidney renal clear cell carcinoma
KIRP	Kidney renal papillary cell carcinoma
LAML	Acute Myeloid Leukemia
LGG	Brain Lower Grade Glioma
LIHC	Liver hepatocellular carcinoma
LUAD	Lung adenocarcinoma
LUSC	Lung squamous cell carcinoma
MESO	Mesothelioma
OV	Ovarian serous cystadenocarcinoma
PAAD	Pancreatic adenocarcinoma
PCPG	Pheochromocytoma and Paraganglioma
PRAD	Prostate adenocarcinoma
SARC	Sarcoma
SKCM	Skin Cutaneous Melanoma
STAD	Stomach adenocarcinoma
TGCT	Testicular Germ Cell Tumors
THCA	Thyroid carcinoma
THYM	Thymoma
UCEC	Uterine Corpus Endometrial Carcinoma
UCS	Uterine Carcinosarcoma
UVM	Uveal Melanoma

Supplementary Table 2. Essential gene enrichment				
**Cluster**	**Gene**	**Gene type**	**CommonEssentialGene_CRISPR/Cas9(DepMap)**	**EssentialGene_Marcotte_2013**
C1	KANSL1	TSG	Yes	
C1	SETD2	TSG	Yes	
C1	RBM10	TSG	Yes	
C1	BRCA1	TSG	Yes	
C1	STAG2	TSG		Yes
C1	SMC1A	OG	Yes	
C1	CHD4	OG	Yes	Yes
C1	RAC1	OG	Yes	
C1	SF3B1	OG	Yes	Yes
C2	BAP1	TSG	Yes	
C4	CUL3	TSG	Yes	
C4	PPP2R1A	OG	Yes	

Supplementary Figures

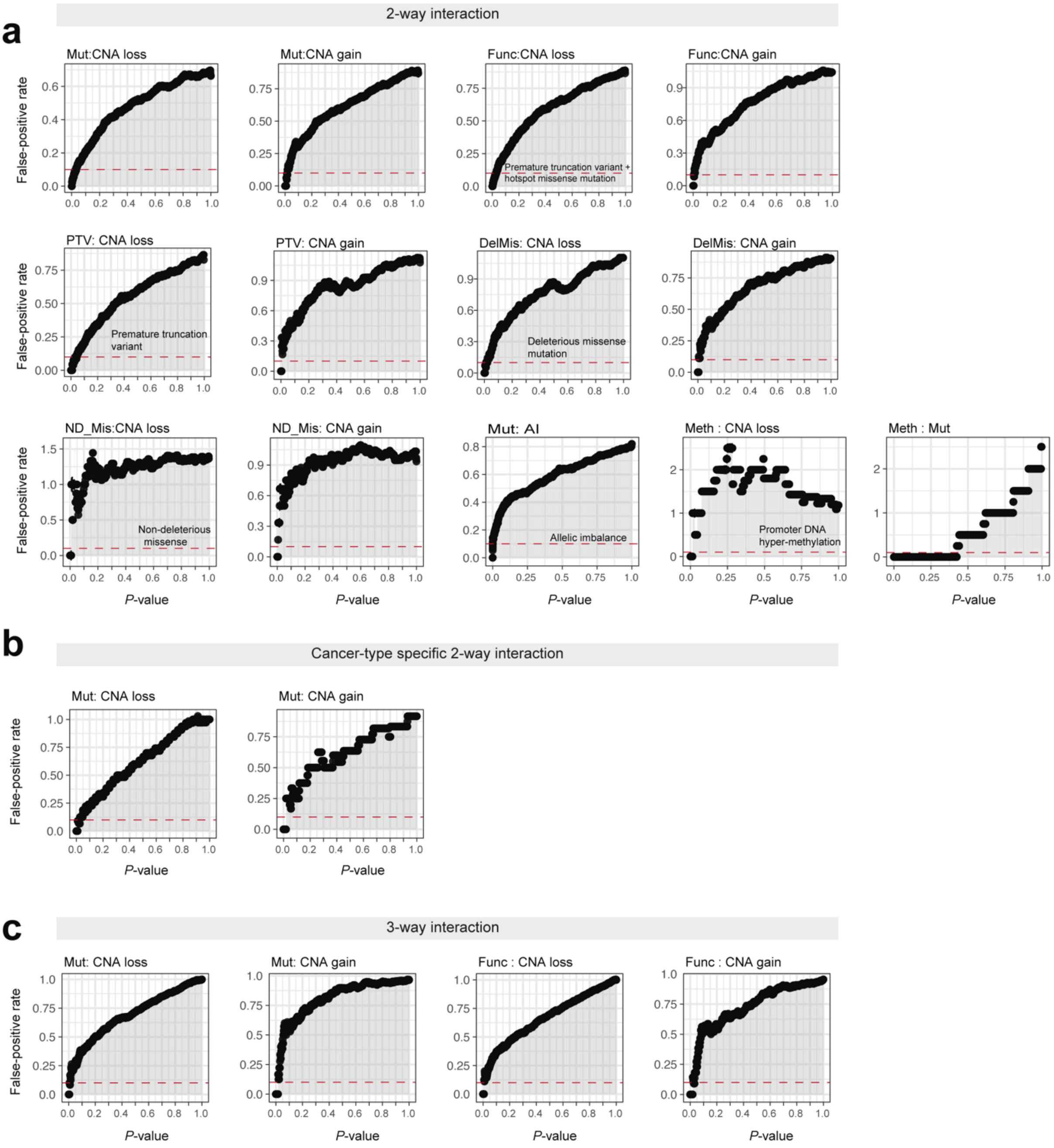

Supplementary Fig. 1: False-positive discovery (FDR) estimates for different p-value thresholds using permutation to identify (**a**) significant 2-way interactions in CNAs loss or gain model, (**b**) cancer-type specific 2-way interaction and (**c**) 3-way interactions from 2-way Mut:CNA loss or gain model. Permutation strategy was applied to determine the significance of the co-occurrence of a pair of two genomic alterations in one gene in the same gene (2-way interaction) or three genomic alterations between two genes (3-way interaction) that controls for the heterogeneity in genomic alterations within and across samples. It maintains the total number of alterations for each genomic alterations across samples as well as the total number of alterations per sample. Each genomic events (mutation, CNA-

loss and CNA-gain) treat them as separate classes and it was performed within each cancer type separately. With 100 permutations, FDR is estimated as the ratio between the number of detected interactions in the permutated matrix and the number of detected with the input matrix in each p-value cut-off. Median FDR values are plotted in each p-value cut-off. Error bars indicate the standard error of the median FDR values.

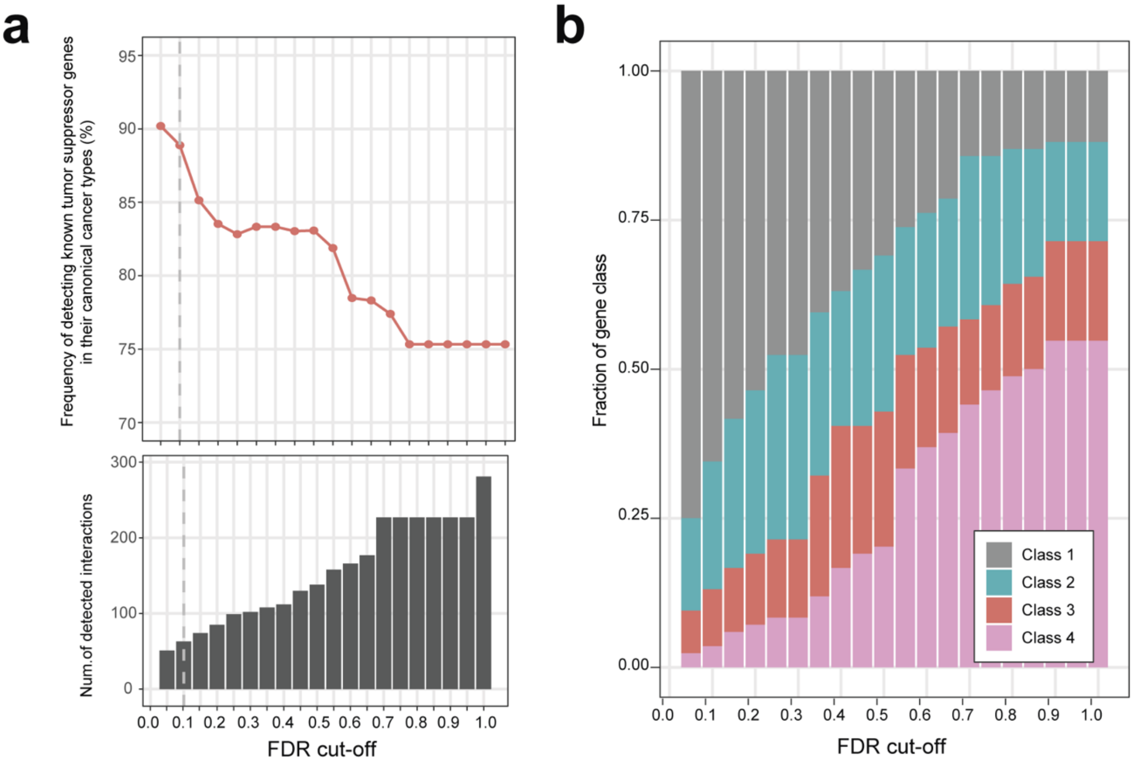

Supplementary Fig. 2: Cancer gene classification depending on FDR cut-off. (**a**) Frequency of detecting known tumor suppressor genes (TSGs) in their canonical cancer types from 281 total number of known TSGs-canonical cancer types pairs in mutation-CNA loss model (top) and number of detected interactions in mutation-CNA loss model (bottom). (**b**) The fraction of different classes of cancer genes in the detected gene-tissue pairs at varying FDR cut-offs.

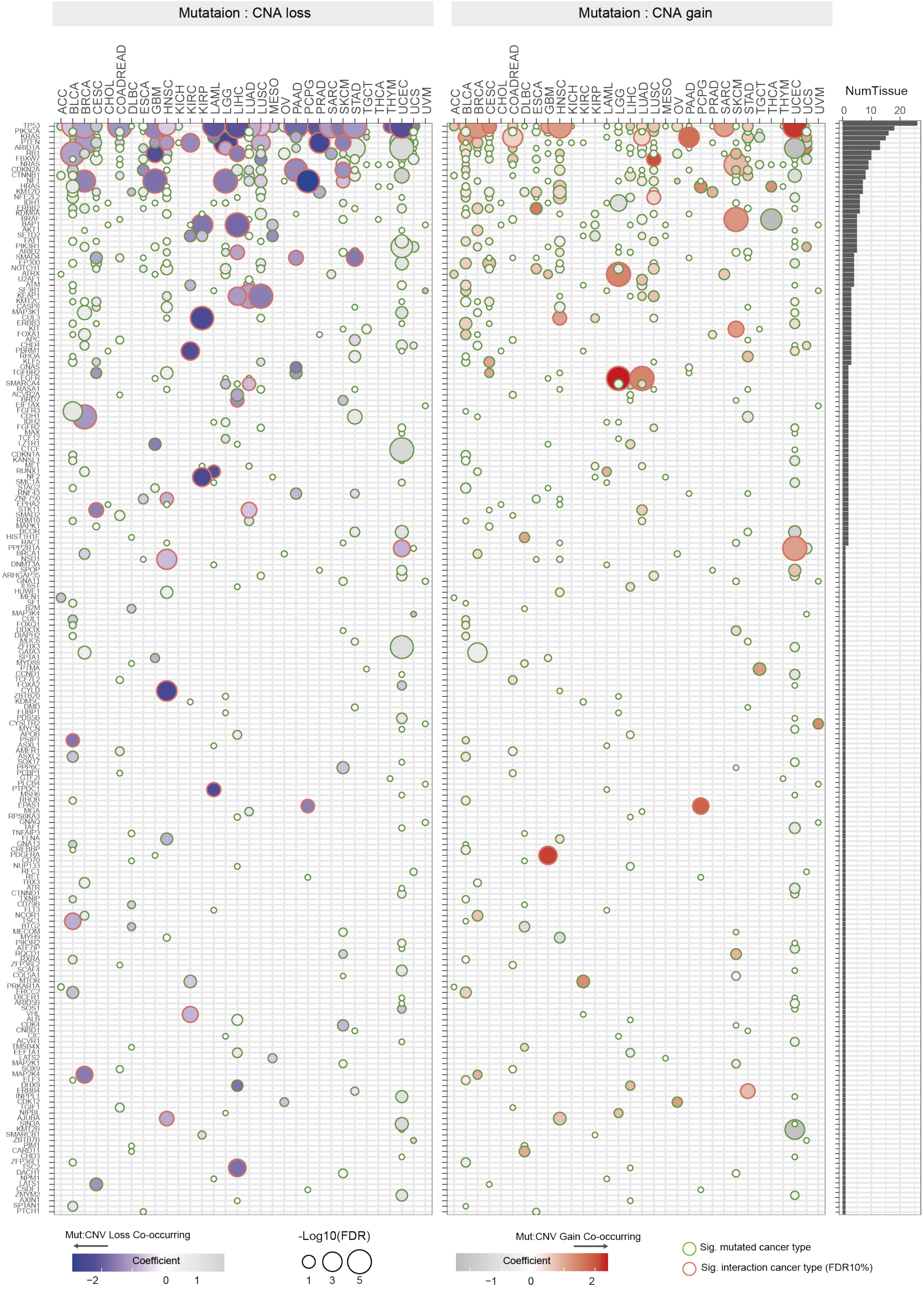

Supplementary Fig. 3: Interactions between somatic mutations and copy-number alterations (CNAs) of all tested cancer genes across cancer types (*N*=201). Coefficient (i.e., effect size) and FDR values

are shown in each cancer type for all tested genes in each cancer type. Color represents the strength of association between the mutation and CNAs within a gene-tissue pair (blue: co-occurrence in the mutation-CNA loss model, red: co-occurrence in the mutation-CNA gain model) and circle size indicates the significance (FDR; -Log10 FDR). The number of tested cancer types in each gene (right) is presented in the bar plot.

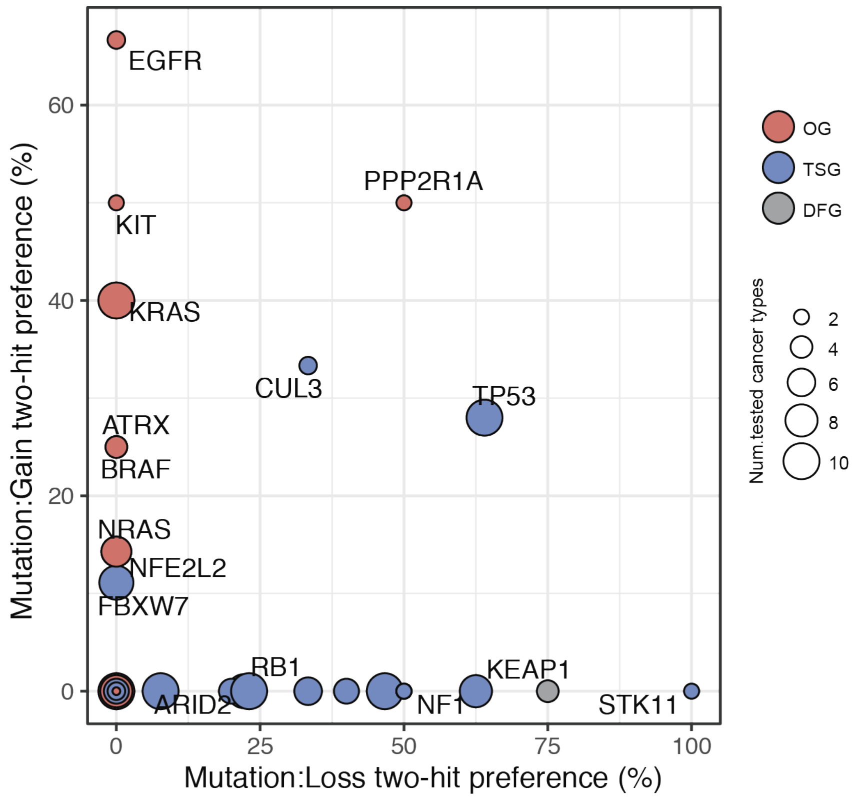

Supplementary Fig. 4: Frequency of interaction (FDR=10%) between mutation and CNA loss (x-axis) and CNA gain (y-axis) in all cancer types in which each gene is mutated in >2% of samples. Color indicates the type of cancer genes (blue: tumor suppressor gene, red: oncogene, grey: dual-functional gene).

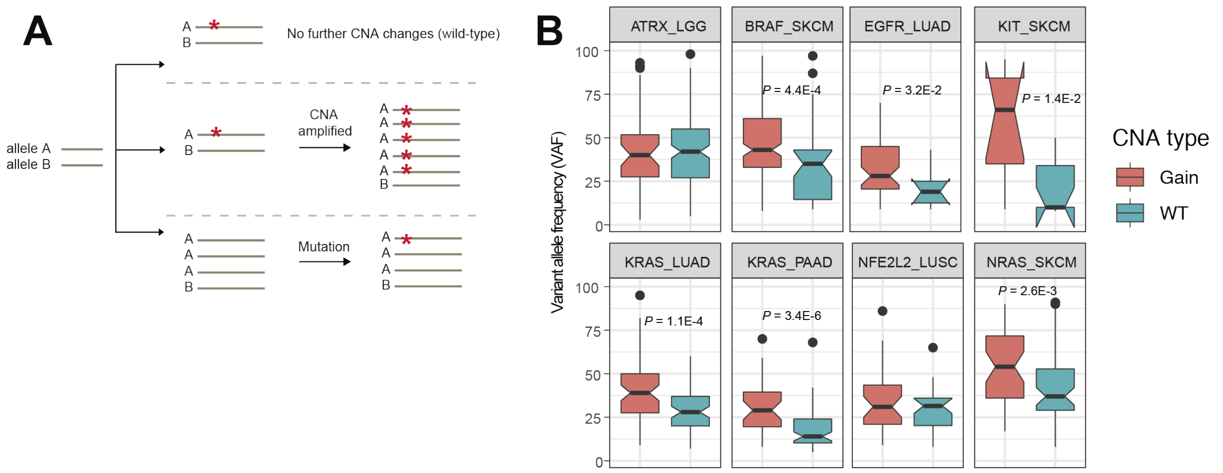

Supplementary Fig. 5: Selection for mutant allele. (**a**) Models of somatic mutation (red star) with any further genomic alteration (upper panel), followed by copy-number amplification (middle panel) and copy-number amplified followed by mutation (bottom panel). (**b**) Significant 2-way interaction pairs (N= 8) in CNA gain model with > 5 samples in both copy-number gain and copy-number wild-type are shown (FDR = 10%; *P*-values between two sample groups from two-sided Mann-Whitney *U*-test). The median value of each gene set is displayed as a band inside each box. The length of each whisker is 1.5 times the interquartile range (shown as the height of each box).

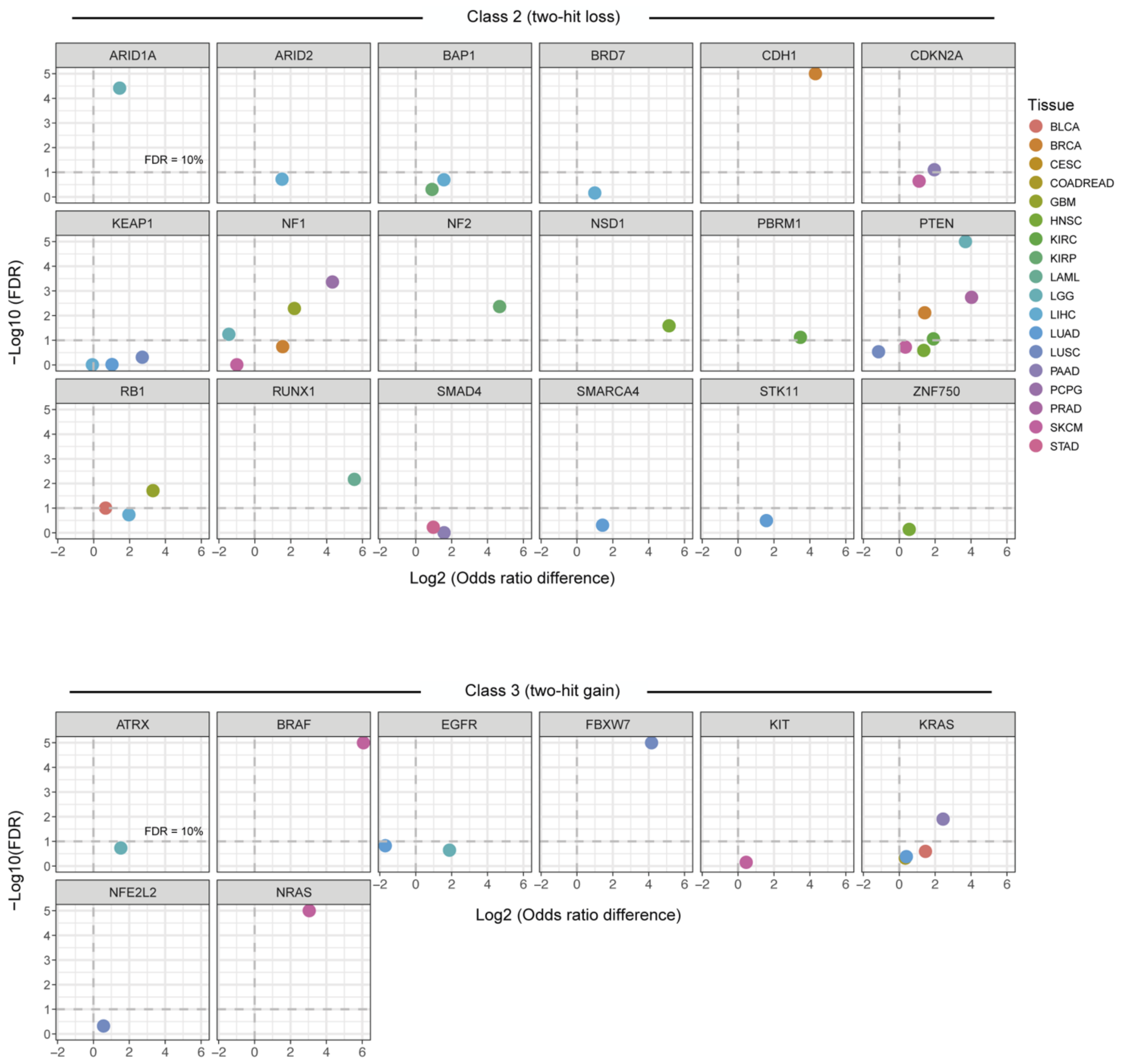

Supplementary Fig. 6: Volcano plot comparing differences of the log of the odds ratios for the co-occurrences between mutation and CNA in two cancer types (i.e., detected cancer type and other significantly mutated cancer types together).

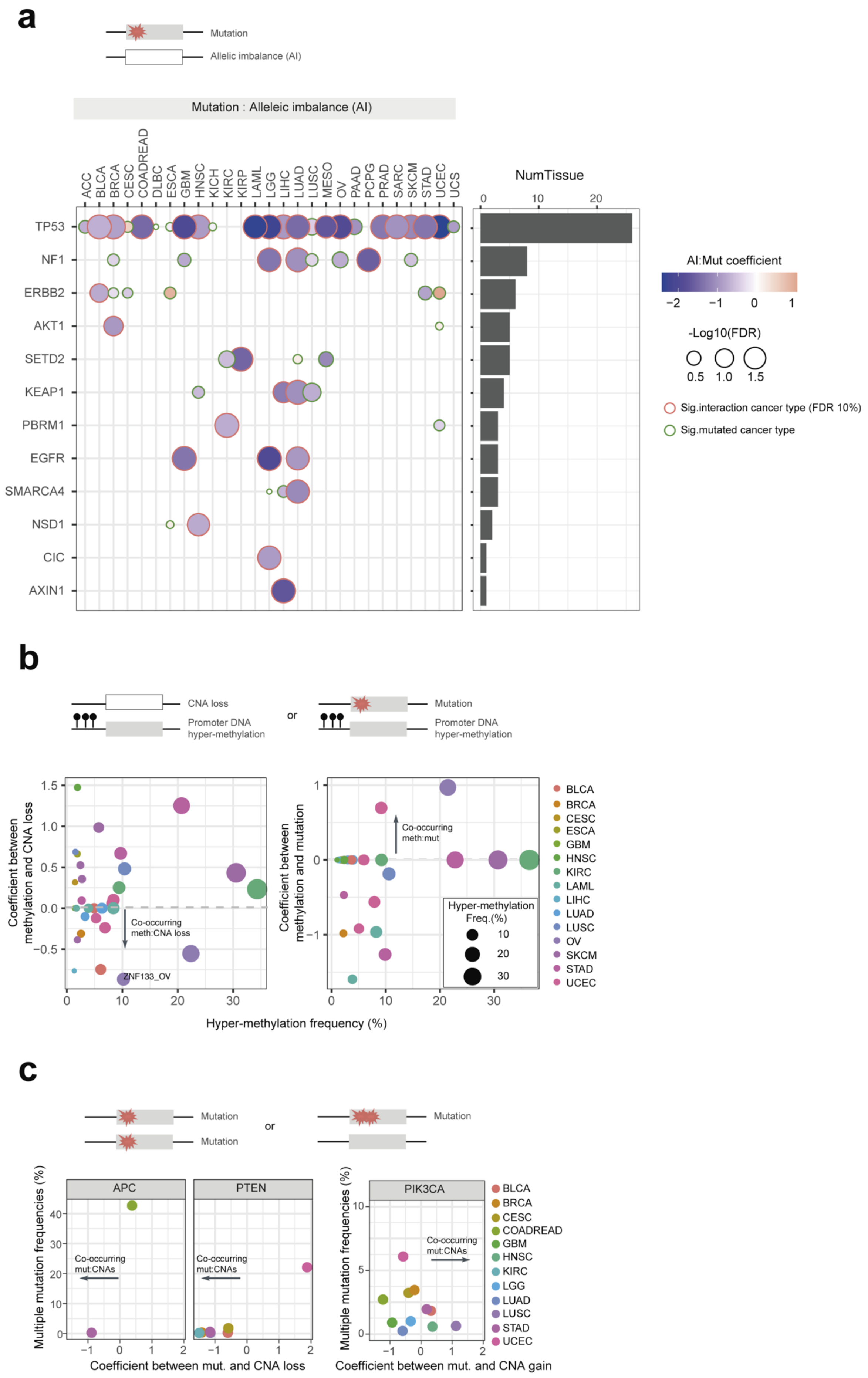
a
Mutation
Allelic imbalance (AI)
Mutation : Alleleic imbalance (AI)
ACC
BLCA
BRCA
CESC
COADREAD
DLBC
ESCA
GBM
HNSC
KICH
KIRC
KIRP
LAML
LGG
LIHC
LUAD
LUSC
MESO
OV
PAAD
PCPG
PRAD
SARC
SKCM
STAD
UCEC
UCS
TP53
NF1
ERBB2
AKT1
SETD2
KEAP1
PBRM1
EGFR
SMARCA4
NSD1
CIC
AXIN1
NumTissue
0
10
20
AI:Mut coefficient
−2
−1
0
1
-Log10(FDR)
0.5
1.0
1.5
Sig.interaction cancer type (FDR 10%)
Sig.mutated cancer type
b
CNA loss
Promoter DNA hyper-methylation
or
Mutation
Promoter DNA hyper-methylation
Coefficient between methylation and CNA loss
Co-occurring meth:CNA loss
ZNF133_OV
Coefficient between methylation and mutation
Co-occurring meth:mut
Hyper-methylation Freq.(%)
10
20
30
Hyper-methylation frequency (%)
BLCA
BRCA
CESC
ESCA
GBM
HNSC
KIRC
LAML
LIHC
LUAD
LUSC
OV
SKCM
STAD
UCEC
c
Mutation
Mutation
or
Mutation
APC
PTEN
PIK3CA
Mutiple mutation frequencies (%)
Co-occurring mut:CNAs
Coefficient between mut. and CNA loss
Coefficient between mut. and CNA gain
BLCA
BRCA
CESC
COADREAD
GBM
HNSC
KIRC
LGG
LUAD
LUSC
STAD
UCEC

Supplementary Fig. 7: Alternative epigenetic and genetic second hits. (**a**) Interactions between allelic imbalance (AI; see Methods) and mutation across cancer types. Coefficient (i.e., effect size) and FDR values are shown in each cancer type for all tested genes in each cancer type. Color represents the strength of association between the mutation and AI within a gene-tissue pair (blue: co-occurrence in the mutation-AI model) and circle size indicates the significance (FDR; -Log10 FDR). The number of tested cancer types in each gene (right) is presented in the bar plot. (**b**) Promoter DNA hyper-methylation frequency (x-axis) plotted against the coefficient for the interaction between hyper-methylation and CNA loss (left) or between hyper-methylation and mutation across gene-tissue pairs. Colors indicate different cancer types and size represents the frequency of hyper-methylation. Significantly interacting pairs are denoted (FDR 10%). (**c**) Frequency of multiple driver mutations (MMs) (x-axis) plotted against the coefficient of co-occurrence between mutation and CNAs loss for tumor suppressor gene pairs (left panel) or CNAs gain for oncogene pairs (right panel). Colors indicate different cancer types.

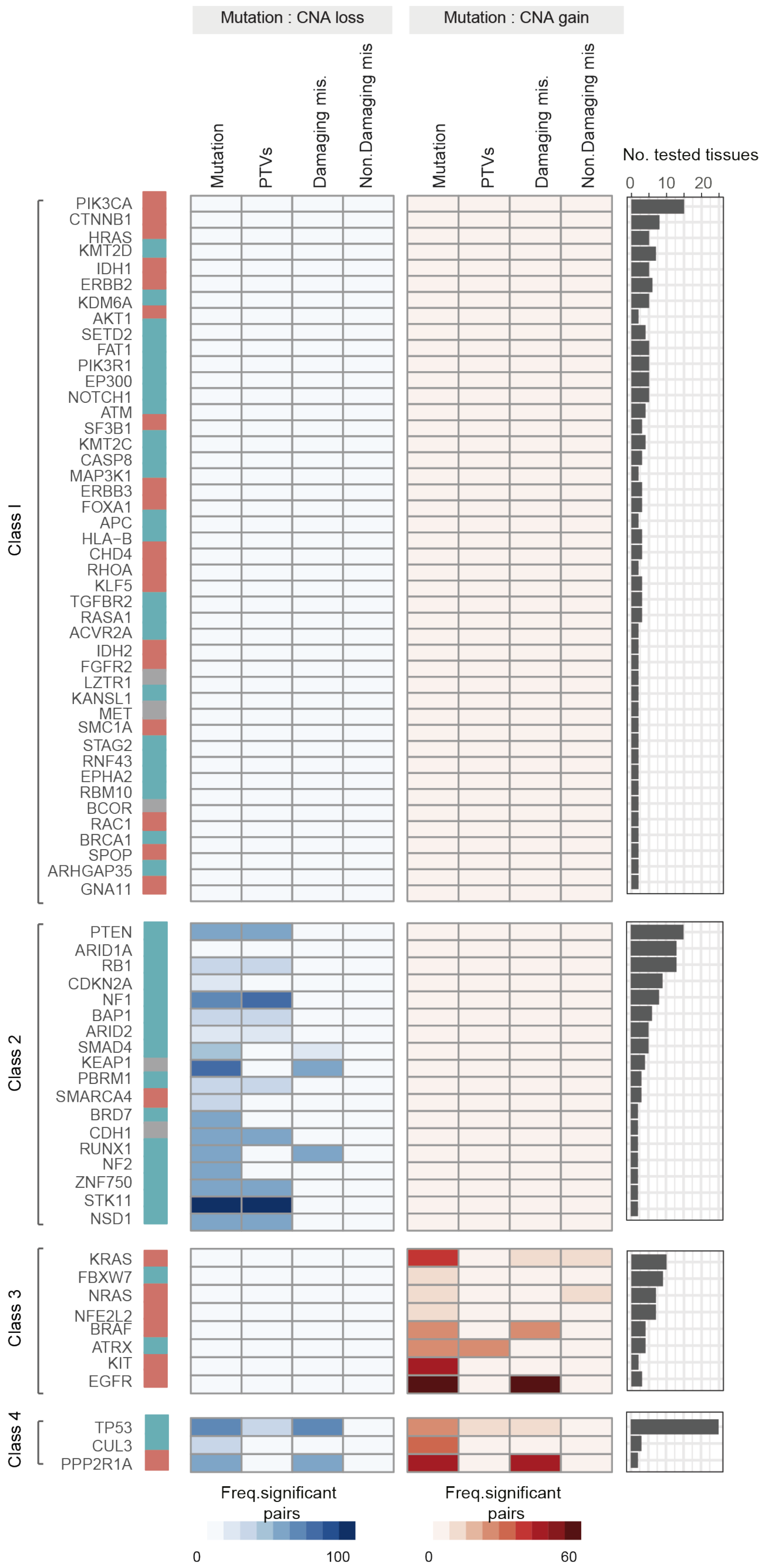

Supplementary Fig. 8: Interaction between mutations and CNAs across different somatic mutation types. Frequencies of detected associations between mutation – CNA loss (left panel) or gain (right

panel) normalized by number of tested tissues for each gene presented across three somatic mutation types: protein-truncation variant, damaging missense mutation and non-damaging missense mutation.

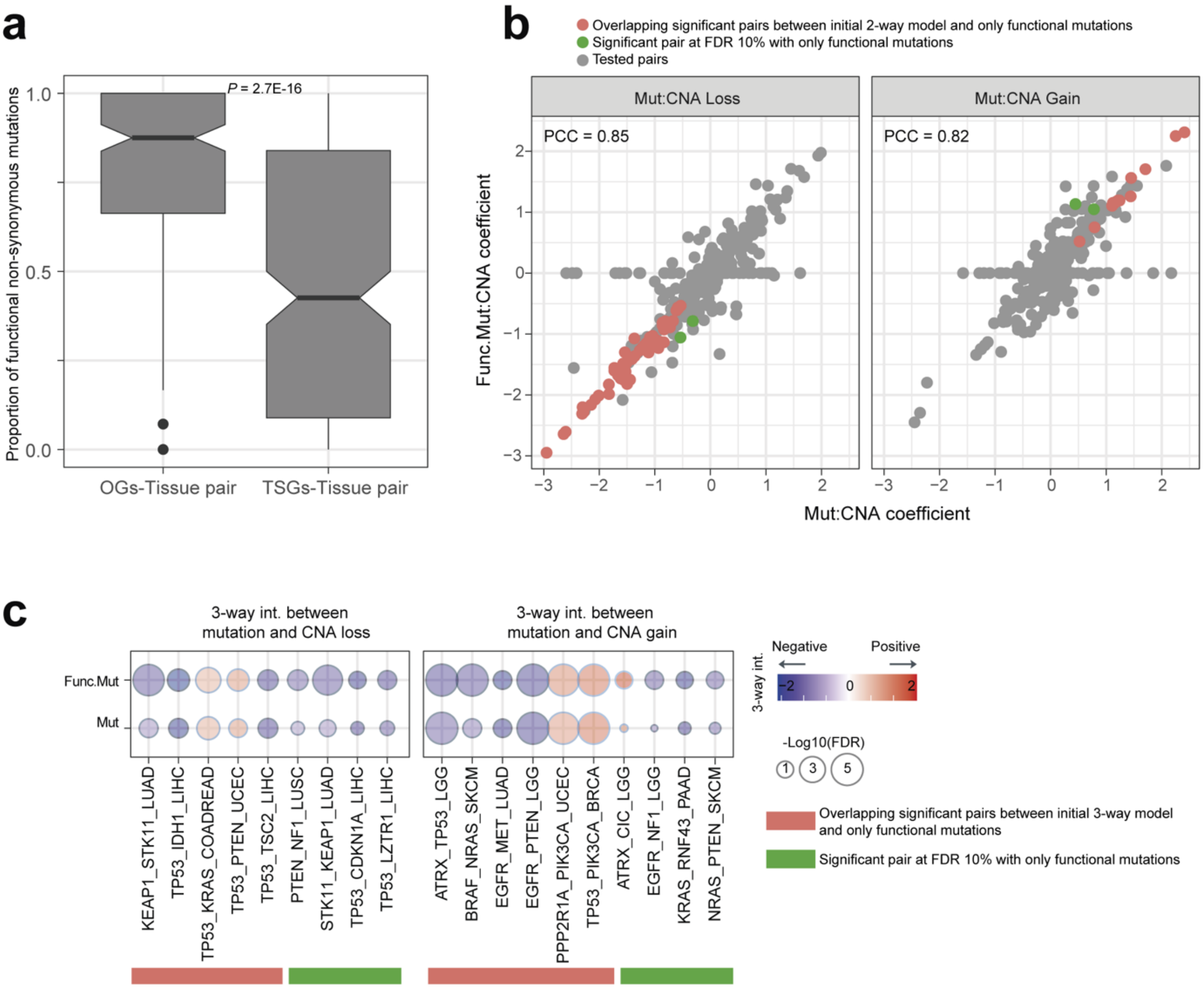

Supplementary Fig. 9: Effect of functional non-synonymous mutations in 2-way and 3-way models. (**a**) The distribution of the proportion of functional non-synonymous mutations between tumor suppressor gene (TSG; N = 252) cancer-type pair and oncogene (OG; N = 204) cancer-type pair (*P*-values between two groups from two-sided Mann-Whitney *U*-test). Functional non-synonymous mutation was defined when recurrently detected the same amino acid position or having evidence of their functional role (Mina et al., 2020). The median value of each gene set is displayed as a band inside each box. The length of each whisker is 1.5 times the interquartile range (shown as the height of each box). (**b**) Coefficient size comparison between initial 2-way models (x-axis; 2-way interaction between PTVs + all non-synonymous mutations and CNAs) *versus* PTV+only functional non-synonymous mutation models (Func.Mut, y-axis; 2-way interaction between PTVs + only functional non-synonymous mutations and CNAs). The Pearson correlation coefficient (PCC) is shown (*P*-value = 2E-16). (**c**) Coefficient size comparison between initial 3-way interactions versus only Func.Mut 3-way interactions (FDR 10%).
