# Supplementary material for: Higher order genetic interactions switch cancer genes from two-hit to one-hit drivers
Source: Nat Commun. 2021 Dec 3;12:7051. doi: 10.1038/s41467-021-27242-3 (PMC8642467; doi:10.1038/s41467-021-27242-3)
Supplement: Supplementary file 10 — Reporting Summary [file 41467_2021_27242_MOESM10_ESM.pdf]

nature research

Corresponding author(s): Ben Lehner

Last updated by author(s): Oct 25, 2021

Reporting Summary

Nature Research wishes to improve the reproducibility of the work that we publish. This form provides structure for consistency and transparency in reporting. For further information on Nature Research policies, see our Editorial Policies and the Editorial Policy Checklist.

Statistics

For all statistical analyses, confirm that the following items are present in the figure legend, table legend, main text, or Methods section.

n/a	Confirmed	
☐	☒	The exact sample size (*n*) for each experimental group/condition, given as a discrete number and unit of measurement
☐	☒	A statement on whether measurements were taken from distinct samples or whether the same sample was measured repeatedly
☐	☒	The statistical test(s) used AND whether they are one- or two-sided<br>*Only common tests should be described solely by name; describe more complex techniques in the Methods section.*
☐	☒	A description of all covariates tested
☐	☒	A description of any assumptions or corrections, such as tests of normality and adjustment for multiple comparisons
☐	☒	A full description of the statistical parameters including central tendency (e.g. means) or other basic estimates (e.g. regression coefficient) AND variation (e.g. standard deviation) or associated estimates of uncertainty (e.g. confidence intervals)
☐	☒	For null hypothesis testing, the test statistic (e.g. F, t, r) with confidence intervals, effect sizes, degrees of freedom and P value noted<br>*Give P values as exact values whenever suitable.*
☒	☐	For Bayesian analysis, information on the choice of priors and Markov chain Monte Carlo settings
☒	☐	For hierarchical and complex designs, identification of the appropriate level for tests and full reporting of outcomes
☐	☒	Estimates of effect sizes (e.g. Cohen's *d*, Pearson's *r*), indicating how they were calculated

Our web collection on statistics for biologists contains articles on many of the points above.

Software and code

Policy information about availability of computer code

Data collection	No new data were collected in this study.
Data analysis	All statistical analyses were carried out using Python 2.7(packages Stats = 1.2.1, NumPy = 1.16.5). Permutation analysis was done using vegan package (2.5.7) in R. Plots were carried out using R version 3.6.2. Packages used for plotting using ggplot2 (version 3.3.3) and pheatmap (version 1.0.12). The custom scripts for regression analysis using R version 3.6.2 can be found here: https://github.com/SolipParkLab/CancerFitness/tree/main/Rcode. Packages for the regression analysis using MASS (version 7.3.53.1), stringr (version 1.4.0), reshape2 (version 1.4.4), data.table (version 1.14.0), dplyr (version 1.0.5), viridis (version 0.5.1).

For manuscripts utilizing custom algorithms or software that are central to the research but not yet described in published literature, software must be made available to editors and reviewers. We strongly encourage code deposition in a community repository (e.g. GitHub). See the Nature Research guidelines for submitting code & software for further information.

Data

Policy information about availability of data

All manuscripts must include a data availability statement. This statement should provide the following information, where applicable:

- Accession codes, unique identifiers, or web links for publicly available datasets
- A list of figures that have associated raw data
- A description of any restrictions on data availability

This study re-analyzed published data sets, including tumor data sets from TCGA Pan-Cancer Atlas. The TCGA somatic mutation data (mc3.v0.2.8.PUBLIC.maf.gz) was downloaded from https://gdc.cancer.gov/about-data/publications/pancanatlas and copy-number alteration data was downloaded from Synapse (syn5049520). Gene Ontology (GO molecular function and biological process) was downloaded from the DAVID 6.8 (https://david.ncifcrf.gov/) and list of Reactome pathways was downloaded from the https://reactome.org/. A list of the somatic driver genes was compiled from Bailey et al (https://doi.org/10.1016/j.cell.2018.02.060).

Epigenetic silencing data was obtained from Saghafinia et al (https://doi.org/10.1016/j.celrep.2018.09.082), multiple driver mutations (MMs) in cancer genes were obtained from Saito et al (https://doi.org/10.1038/s41586-020-2175-2), and allelic imbalance data were obtained from Park et al (https://doi.org/10.1038/s41467-018-04900-7). A list of functional non-synonymous mutations was collected from Mina et al (https://doi.org/10.1038/s41588-020-0703-5). Source data are provided with this paper.

Field-specific reporting

Please select the one below that is the best fit for your research. If you are not sure, read the appropriate sections before making your selection.

☒ Life sciences ☐ Behavioural & social sciences ☐ Ecological, evolutionary & environmental sciences

For a reference copy of the document with all sections, see nature.com/documents/nr-reporting-summary-flat.pdf

Life sciences study design

All studies must disclose on these points even when the disclosure is negative.

Sample size	Sample sizes were not predetermined as we aimed to use all possible samples available on TCGA based on TCGA guidelines. Total number of TCGA samples in this study is 9,175 across 33 cancer types.
Data exclusions	From all available TCGA samples, we initially selected samples after removing hyper-mutated samples and excluding them by pathology review. Next, we limited our analyses to samples that have information both in somatic mutations and copy-number alterations.
Replication	Replication is not applicable, since there was no experimental data.
Randomization	To determine the significance of the co-occurrence of a pair of two genomic alterations (somatic mutation and copy-number alteration within a gene), we applied a permutation strategy that controls for the mutational heterogeneity within and across tumor samples. We used the permatswap function in the R package vegan (http://vegan.r-forge.r-project.org/) to produce permutated genomic alteration matrices that maintain the total number of alterations for each alteration across samples as well as the total number of alterations per sample, considering copy number alterations and somatic mutations. It means the total number of genomic alterations across individual events and individual samples are the same as the input matrix but the co-occurrence between two genomic alterations can be changed by randomly assigned genomic alteration events. We permuted genomic events for each cancer type separately to control for any biases in alteration frequencies in the different cancer types. A total of 100 permutations were performed.
Blinding	Blinding was not relevant to the study. There were no treatment and control groups.

Reporting for specific materials, systems and methods

We require information from authors about some types of materials, experimental systems and methods used in many studies. Here, indicate whether each material, system or method listed is relevant to your study. If you are not sure if a list item applies to your research, read the appropriate section before selecting a response.

Materials & experimental systems

n/a	Involved in the study	
☒	☐	Antibodies
☒	☐	Eukaryotic cell lines
☒	☐	Palaeontology and archaeology
☒	☐	Animals and other organisms
☒	☐	Human research participants
☒	☐	Clinical data
☒	☐	Dual use research of concern

Methods

n/a	Involved in the study	
☒	☐	ChIP-seq
☒	☐	Flow cytometry
☒	☐	MRI-based neuroimaging
